# Evaluation of risk factors’ importance on adverse pregnancy and neonatal outcomes in women aged 40 years or older

**DOI:** 10.1186/s12884-019-2239-1

**Published:** 2019-03-13

**Authors:** Gunilla Sydsjö, Malin Lindell Pettersson, Marie Bladh, Agneta Skoog Svanberg, Claudia Lampic, Elizabeth Nedstrand

**Affiliations:** 1 Department of Obstetrics and Gynaecology, and Department of Clinical and Experimental Medicine, Linköping University, Linköping, Sweden. Linköping University, SE-581 85 Linköping, Sweden; 2 Department of Women’s and Children’s Health, Uppsala University, Uppsala, Sweden; 3 Department of Neurobiology, Care Sciences and Society, Karolinska Institutet, S-141 83 Huddinge, Sweden; 4 Department of Women’s and Children’s Health, Karolinska Institutet, S-171 77 Stockholm, Sweden

**Keywords:** Advanced maternal age, Pregnancy, Delivery, Neonate status

## Abstract

**Background:**

Women of advanced age (40 years or older) are generally, at risk for pregnancy and delivery related problems. In addition, there is limited knowledge on being of advanced age and having been given Assisted Reproductive Treatment (ART) and its association with negative obstetric outcomes. Therefore, data from the Swedish Medical Birth Register was used to investigate pregnancy and neonatal outcomes for women aged 40 or more who had given birth. The secondary aim was to compare the obstetric outcomes of women who had used ART and women who had not **undergone** ART while adjusting for marital status across the age groups.

**Method:**

Women of advanced age who had given birth in Sweden during 2007–2012 formed the index group, *n* = 37,558; a reference group of women comprised 71,472 women under the age of 40. An additional subgroup of women aged 45 or older when giving birth was also formed, *n* = 2229. The obstetric and neonatal data for all the women was derived from national register data.

**Results:**

Women of advanced age were more often single, had **undergone** ART, and more often experienced adverse obstetric outcomes than did younger women. The neonate’s health was also more often adversely affected expressed as being **born with low birth weight** and Small for Gestational Age (SGA), having lower Apgar scores, and having more health problems during the first week compared to the reference group.

**Conclusions:**

Women who are approaching the upper limit of fecundity are at greater risk for having children who are preterm and SGA. The adverse effects of being preterm and SGA may have negative long-term effects, not only on the children but also on the mothers. This needs to be addressed more frequently in a clinical setting when advising women of all ages on pregnancy and ART treatment.

## Background

During the past three decades there has been an increase in the percentage of women in the Western world who delay childbirth [1, 2]. The percentage of women over 40 and even 45 years of age, who have given birth, has been increasing steadily. In Sweden, the percentage of women who are childless until they are 35 years or older has increased four-fold between 1984 and 2014 [3, 4]; during the same period the number of first-time mothers over 40 years of age increased from 170 to 1199, i.e. a seven-fold increase [3]. The use of assisted reproductive technology (ART) in the form of gamete donation, embryo donation and In Vitro Fertilization (IVF), is an available choice in many countries and the possibilities for older women to become pregnant and to form a family have therefore become greater.

Women giving birth at an advanced age, defined as age 40 or older, are at an increased risk of complications during pregnancy and delivery, such as gestational diabetes and preeclampsia, compared to younger women [5]. They are also more prone to having children with a low or high birth weight [6], a caesarean delivery compared to younger women [7]. Moreover, their children are more often born moderately or very preterm, small for gestational age (SGA), with low Apgar scores, exhibiting fetal distress and even fetal and neonatal death [5, 7–12]. The risks associated with pregnancy and delivery in women of advanced age tend to remain higher than for younger women even after adjusting for socio-demographic factors [7].

In Sweden, couples and single women can use ART to conceive, several different options are available including IVF with their own gametes or oocyte−/sperm- donation, until the age of 42. Embryo donation was prohibited until the end of 2018 and treatment of single women has only recently been allowed [13]. Because of these restrictions, some Swedish women and couples have travelled abroad to have embryo-, oocyte- and/or sperm- donation. We have little knowledge about pregnancy and delivery outcomes for older Swedish women, those who are aged 40 or older and have received ART. In Sweden most IVF clinics do not provide ART for this group of women, so many women in this group who have used ART have done so outside of Sweden. However, several studies have shown that increasing maternal age as well as being single increases the risk for adverse pregnancy and delivery outcomes [14–16]. Also, using ART to achieve pregnancy has been shown to increase the risk for adverse pregnancy and delivery outcomes [15]. Thus, one can hypothesize that using ART to achieve pregnancy for older single woman may impose even greater risks for adverse outcomes.

Thus, the primary aim of this study was to investigate pregnancy and neonatal outcomes for women of advanced age. The secondary aim was to compare the obstetric outcomes of women who had used ART and women who had not used ART while adjusting for marital status across the age groups.

## Methods

This study was designed as a case control study where data were obtained from the Medical Birth Register (MBR). As there is evidence in the literature that women at or over 40 years of age constitute an obstetric risk group, we chose this age cut-off for defining women of advanced age. Women 40 years of age or older who gave birth in Sweden between 2007 and 2013 formed the index group, *n* = 37,558. This group was compared to a matched control group of women under the age of 40 who also gave birth during the same time period, *n* = 71,472. The matching criteria were parity and year of birth. A subgroup of women aged 45 years or older when they gave birth was also formed, *n* = 2229 (of 37,558, 5.9%). The only exclusion criterion for this study was that none of the index women could serve as a control to themselves.

### The Swedish medical birth register

The Swedish Medical Birth Register (MBR) is held by the Swedish National Board and Health and Welfare and contains medical information on practically all deliveries in Sweden from 1973 until the present [17].

### Measures

This study used several different measures that can be divided into three main categories: socio-demographic and medical data (including civil status, employment, tobacco use, BMI, chronic illness, parity, and use of ART), pregnancy and delivery data (pre-eclampsia/eclampsia, gestational diabetes, gestational hypertension, premature contractions, infections during pregnancy, haemorrhage pre-, intra- and post-partum, mode of delivery, and anaesthetic used during delivery) and neonatal data (birth weight, twinning, gestational age, size for gestational age, Apgar, child’s overall health at birth and survival rate first month). The two latter categories constituted the outcome measures of interest. Definitions of all variables included in the study can be found in Table 1.

**Table 1:** Variable definitions

Variable	Categories
Maternal age	≤39 years of age, 40–44 years of age, ≥45 years of age
Civil status	Married/cohabiting, Single
Employment	Unemployed/student/other, Full time employment, Part time employment
Maternal BMI	Normal, Underweight, Overweight, Obese
Tobacco use	No, Yes
Chronic illness	No, Yes
ART	No, Yes
Pre-eclampsia/eclampsia	No, Yes
Gestational diabetes	No, Yes
Gestational hypertension	No, Yes
Premature contractions	No, Yes
Infection during pregnancy	No, Yes
Haemmorhage (pre-, intra-, and post-partum)	No, Yes
Paracervical block	No, Yes
Pethidine	No, Yes
Pudendal block	No, Yes
Epidural	No, Yes
Mode of delivery	Normal delivery, Elective caesarean, Emergency caesarean, Instrumental delivery
Gestational age	Very preterm, < 32 weeks, Preterm, 32–36 weeks, At term, 37–42 weeks, Post term, > 42 weeks
Birthweight	Normal birthweight, > = 2500 g, Low birthweight, 1500-2499 g, Very low birthweight, < 1500 g
Size for gestational age	Appropriate for gestational age (AGA), Small for gestational age (SGA), Large for gestational age (LGA)
Apgar, 5 min	0–6, 7–10
Apgar, 10 min	0–6, 7–10
Child’s health at delivery	Healthy – Yes, Healthy – No
Neonatal survival	0–27 days, > = 28 days

### Statistical analysis

The relationship between age when becoming a mother and socio-demographic data, pregnancy, delivery data, as well as neonatal data was initially analyzed using Pearson’s Chi-square statistic and Student’s t-test for continuous outcomes. Multivariate analyses included linear regression for continuous outcomes, such as gestational age, birthweight and the child’s length, while multiple logistic regression was used for dichotomous outcomes (presence of pre-eclampsia/eclampsia, gestational diabetes, gestational hypertension, healthy child, survival during the first four weeks). In addition, data was stratified by age groups in order to analyze the relationship between type of pregnancy (ART vs. spontaneous) and civil status, gestational age, birthweight, and size for gestational age. All analyses were adjusted for civil status, mother’s BMI, and indicator for ART/ not ART. In most of the variables some partial missing data was present. Therefore, in the analyses only data with complete observations have been included. A *p*-value < 0.05 (two-sided) was considered statistically significant. All statistical analyses were performed using IBM SPSS, version 23 (IBM SPSS Inc., Armonk, NY).

### Ethical approval

This study was approved by the Regional Ethical Review Board, Linköping, Sweden and was performed according to the Declaration of Helsinki. No 2014/111–31. Date: 26-03-2016.

## Results

The older the women were the more likely they were to have been single at the time of the child’s birth. Women between 40 and 44 years of age were 50% more likely to be single, while women aged 45 years or more were twice as likely to be single compared to women younger than 40. Women of advanced age were also more likely to be overweight or obese, and to have conceived using ART, Table 2. Moreover, the likelihood for having either pre-eclampsia/eclampsia or gestational diabetes increased with the mother’s age when giving birth, Table 2. Also, with increasing age the proportion of women having an elective caesarean section increased from 7.5% among the youngest (below 40 years of age) to 20.4% among the oldest (45 years or older), Table **3**.

**Table 2:** Socio-demographic and medical background data on the study population^a^

	Mothers ≤ 39 *n* = 71,472		Mothers 40–44 *n* = 35,329		Mothers ≥ 45 *n* = 2229		*P*-value^1^	*P*-value^2^	*P*-value^3^	*P*-value^4^
	min-max (mean/sd)		min-max (mean/sd)		min-max (mean/sd)					
Maternal age, min-max (mean/sd)	14–39 (31.45/4.54)		40–44 (41.41/1.19)		45–55 (45.92/1.39)		< 0.001	< 0.001	< 0.001	< 0.001
	n	%	n	%	n	%	*P*-value^1^	*P*-value^2^	*P*-value^3^	*P*-value^4^
Civil status							< 0.001	< 0.001	< 0.001	< 0.001
Married/ cohabiting	64,059	89.6	30,106	85.0	1802	80.8				
Single status at registration	7413	10.4	5323	15.0	427	19.2				
Parity							0.001	< 0.001	0.664	0.126
Primiparous	15,660	21.9	7417	20.9	497	22.3				
Multiparous	55,812	78.1	28,012	79.1	1732	77.7				
Worked during pregnancy							< 0.001	< 0.001	< 0.001	0.004
Unemployed/ student/ other	17,103	27.4	6418	20.8	454	23.9				
Full time	27,543	44.2	16,219	52.5	964	50.8				
Part time	17,717	28.4	8267	26.8	478	25.2				
Tobacco use during pregnancy							< 0.001	< 0.001	0.004	0.383
Yes	8329	12.3	3588	10.8	210	10.2				
Mother’s BMI (registration)							< 0.001	< 0.001	< 0.001	0.003
Normal	35,306	56.2	16,156	53.2	905	48.9				
Underweight	1165	1.9	291	1.0	21	1.1				
Overweight	17,117	27.3	9338	30.8	611	33.0				
Obese	9185	14.6	4566	15.0	314	17.0				
Chronic illness^b^							0.725	0.890	0.440	0.422
Yes	15,035	21.0	7440	21.0	484	21.7				
ART							< 0.001	< 0.001	< 0.001	< 0.001
Yes	1894	2.6	2813	7.9	249	11.2				

^a^Numbers may not add up to the total due to partially missing data

^b^e.g. Diabetes, Hypertension, Arthritis, SLE, Kidney disease, Epilepsy, Asthma, Ulcerative colitis

^1^Across all age groups

^2^≤39 vs. 40–44

^3^≤39 vs. ≥45

^4^40–44 vs. ≥45

**Table 3:** Pregnancy and delivery outcomes for the study population

	Mothers ≤ 39 *n* = 71,472		Mothers 40–44 *n* = 35,429		Mothers ≥ 45 *n* = 2229		*p*-value^1^	*p*-value^2^	*p*-value^3^	*p*-value^4^
	n	%	n	%	n	%				
Pre-eclampsia/eclampsia							< 0.001	< 0.001	< 0.001	< 0.001
Yes	1776	2.5	1365	3.9	146	6.6				
Gestational diabetes							< 0.001	< 0.001	< 0.001	< 0.001
Yes	935	1.3	917	2.6	86	3.9				
Gestational hypertension							< 0.001	< 0.001	< 0.001	0.017
Yes	679	1.0	549	1.5	49	1.2				
Premature contractions							0.008	0.011	0.055	0.169
Yes	229	0.31	82	0.2	2	0.1				
Infection							0.966	0.815	0.925	0.873
Yes	200	0.3	102	0.3	6	0.3				
Haemorrhage, prepartum							0.910	0.525	0.677	0.509
Yes	25	0.0	11	0.0	1	0.0				
Mode of delivery							< 0.001	< 0.001	< 0.001	< 0.001
Caesarean section (elective)	5328	7.5	4931	13.9	455	20.4				
Caesarean section (emergency)	4931	6.9	4249	12.0	370	16.6				
Instrumental delivery^b^	3592	5.0	2255	6.4	118	5.3				
Epidural							< 0.001	< 0.001	< 0.001	0.001
Yes	14,454	20.2	6809	19.2	363	16.3				
Haemorrhage, intrapartum							< 0.001	< 0.001	< 0.001	< 0.001
Yes	1242	1.7	1206	3.4	124	5.5				
Haemorrhage, postpartum							< 0.001	< 0.001	< 0.001	0.010
Yes	3013	4.2	1658	4.7	131	5.9				
Paracervical block ^a^							0.571	0.296	0.803	0.973
Yes	922	1.5	371	1.4	20	1.4				
Pethidine ^a^							0.019	0.013	0.258	0.063
Yes	1738	2.8	667	2.5	47	3.3				
Pudendal block ^a^							< 0.001	< 0.001	0.402	0.617
Yes	1446	2.4	771	2.9	38	2.7				

^a^Women delivered by caesarean section were excluded

^b^Vaginal delivery where either thongs or vacuum extraction was used

^1^Across all age groups, Person’s chi-square

^2^≤39 vs. 40–44, Pearson’s chi-square

^3^≤39 vs. ≥45, Pearson’s chi-square

^4^40–44 vs. ≥45, Pearson’s chi-square

Having a child born either preterm or very preterm was close to being twice as likely among women aged 45 or more compared to women below the age of 40, Table **4**. Women of advanced age were also twice as likely to deliver a twin or a child with low or very low birthweight. Moreover, the oldest mothers were also at a significantly higher risk for delivering a child with low Apgar, at 5 and 10 min, a child who was not well as a neonate, and to have children more likely to die within the first four weeks after delivery, Table **4**.

**Table 4:** Neonatal birth characteristics for the three study groups^a^

	Mothers ≤ 39 *n* = 71,472		Mothers 40–45 *n* = 35,429		Mothers ≥ 45 *n* = 2229		CHI-SQUARE/ANOVA			
	mean/SD		mean/SD		mean/SD		*p*-value^1^	*p*-value^2^	*p*-value^3^	*p*-value^4^
Child’s length, cm (mean/SD)	50.34/ 2.65		50.27/ 2.85		49.85/ 3.30		< 0.001^b^	< 0.001^b^	< 0.001^b^	< 0.001^b^
Child’s weight, gram (mean/SD)	3541.49/ 604.21		3507.06/ 639.68		3403.80/ 706.41		< 0.001^b^	< 0.001^b^	< 0.001^b^	< 0.001^b^
	n	%	n	%	n	%	*p*-value^1^	*p*-value^2^	*p*-value^3^	*p*-value^4^
Twin							< 0.001	0.007	< 0.001	< 0.001
No	68,452	95.8	34,056	96.1	2033	91.2				
Yes	3020	4.2	1373	3.9	196	8.8				
Gestational age							< 0.001	< 0.001	< 0.001	< 0.001
Very preterm, < 32 weeks	680	1.0	436	1.2	54	2.4				
Preterm, 32–36 weeks	3870	5.4	2156	6.1	187	8.4				
Post term, > 42 weeks	245	0.3	127	0.4	8	0.4				
Birthweight							< 0.001	< 0.001	< 0.001	< 0.001
Low birthweight, 1500 g-2499 g	2633	3.7	1558	4.4	153	6.9				
Very low birthweight, < 1500 g	534	0.7	405	1.1	48	2.2				
Size for gestational age							< 0.001	< 0.001	< 0.001	0.884
AGA^5^	67,107	93.9	32,710	92.3	2052	92.1				
SGA^6^	1281	1.8	987	2.8	63	2.8				
LGA^7^	3084	4.3	1732	4.9	114	5.1				
Apgar 5							< 0.001	< 0.001	< 0.001	0.168
Low score, 0–6	763	1.1	546	1.6	43	1.9				
Apgar 10							< 0.001	< 0.001	< 0.001	0.015
Low score0–6	253	0.4	181	0.5	20	0.9				
Child health^c^							< 0.001	< 0.001	< 0.001	< 0.001
Healthy – No	14,282	20.4	8422	24.3	602	27.7				
Survival							< 0.001	< 0.001	0.011	0.328
0–27 days	123	0.2	102	0.3	9	0.4				

^a^Student’s t-test

^b^Numbers may not add up to the total due to partially missing data

^c^Defined as: Healthy child, examined at the delivery ward

^1^Across all age groups

^2^≤39 vs. 40–44, Pearson’s chi-square

^3^≤39 vs. ≥45, Pearson’s chi-square

^4^40–44 vs. ≥45, Pearson’s chi-square

^5^Appropriate for gestational age

^6^Small for gestational age

^7^Large for gestational age

Data were further stratified by method of conception, in order to investigate the differences in birth characteristics and civil status in relation to age when becoming a mother in each of these groups. The analysis showed that in both groups (ART and spontaneous pregnancy) women of advanced age were more prone to be single, delivering a child preterm or with low birthweight as well as an SGA child, compared to women younger than 40 (Table **5**). Furthermore, the multiple logistic regression, where data was stratified by age, it was found that women who had used ART to achieve pregnancy were more likely to be single, and to give birth to a preterm child in all age groups. Moreover, **women** of advanced age had an increased likelihood of delivering a very preterm child (Table **6**).

**Table 5:** Birth characteristics and civil status by maternal age when giving birth and type of pregnancy ^a^

	ART				Spontaneous			
	≤39	40–44	≥45	*p*-value^b^	≤39	40-44	≥45	*p*-value^b^
	*n* (%)	*n* (%)	*n* (%)		*n* (%)	*n* (%)	*n* (%)	
Civil status				< 0.001				< 0.001
Married/ cohabiting	1859 (98.2)	2651 (94.2)	227 (91.2)		62,200 (89.4)	27,455 (84.2)	1575 (79.5)	
Single status at registration	35 (1.8)	162 (5.8)	22 (8.8)		7378 (10.6)	5161 (15.8)	405 (20.5)	
Parity								< 0.001
Primiparous	872 (46.0)	1318 (46.9)	120 (48.2)	0.754	14,788 (21.3)	6099 (18.7)	377 (19.0)	
Multiparous	1022 (54.0)	1495 (53.1)	129 (51.8)		54,790 (78.7)	26,517 (81.3)	1603 (81.0)	
Twin				< 0.001				< 0.001
No	1404 (74.1)	2381 (84.6)	170 (68.3)		67,048 (96.4)	31,675 (97.1)	1863 (94.1)	
Yes	490 (25.9)	432 (15.4)	79 (31.7)		2530 (3.6)	941 (2.9)	117 (5.9)	
Gestational age				< 0.001				< 0.001
At term, 37–42 weeks	1556 (82.2)	2454 (87.2)	190 (76.3)		65,066 (93.6)	30,223 (92.8)	1787 (90.4)	
Post term, > 42 weeks	4 (0.2)	11 (0.4)	1 (0.4)		241 (0.3)	116 (0.4)	7 (0.4)	
Preterm, 32–36 weeks	277 (14.6)	284 (10.1)	49 (19.7)		3593 (5.2)	1872 (5.7)	138 (7.0)	
Very preterm, < 32 weeks	57 (3.0)	64 (2.3)	9 (3.6)		623 (0.9)	372 (1.1)	45 (2.3)	
Birthweight				< 0.001				< 0.001
Normal birthweight, ≥2500 g	1614 (85.5)	2526 (90.0)	193 (77.8)		66,533 (95.8)	30,834 (94.8)	1831 (92.6)	
Low birthweight, 1500 g-2499 g	230 (12.2)	228 (8.1)	45 (18.1)		2403 (3.5)	1330 (4.1)	108 (5.5)	
Very low birthweight, < 1500 g	44 (2.3)	52 (1.9)	10 (4.0)		490 (0.7)	1330 (1.1)	38 (1.9)	
Size for gestational age				0.255				< 0.001
AGA^1^	1788 (94.4)	2637 (93.7)	226 (90.8)		65,319 (93.9)	30,073 (92.2)	1826 (92.2)	
SGA^2^	42 (2.2)	72 (2.6)	10 (4.0)		1239 (1.8)	915 (2.8)	53 (2.7)	
LGA^3^	64 (3.4)	104 (3.7)	13 (5.2)		3020 (4.3)	1628 (5.0)	101 (5.1)	

^a^Numbers may not add up to the total due to partially missing data

^b^Pearson’s chi-square

^1^Appropriate for gestational age

^2^Small for gestational age

^3^Large for gestational age

**Table 6:** Odds ratios for women in the different age groups who had given birth following ART vs. spontaneous pregnancy^a^

	Age group				All age groups
	≤39	40–44	≥45	≥40	
	OR (95% CI)	OR (95% CI)	OR (95% CI)	OR (95% CI)	OR (95% CI)
Civil status					
Married/ cohabiting	*Reference*	*Reference*	*Reference*	*Reference*	*Reference*
Single status at registration	7.51 (5.35–10.53)	4.09 (3.46–4.83)	3.71 (2.28–6.04)	0.73 (3.24–4.29)	4.62 (4.02–5.33)
Parity					
Primiparous	*Reference*	*Reference*	*Reference*	*Reference*	*Reference*
Multiparous	0.27 (0.25–0.30)	0.21 (0.20–0.23)	0.22 (0.16–0.30)	0.25 (0.24–0.27)	0.24 (0.22–0.25)
Twin					
No	*Reference*	*Reference*	*Reference*	*Reference*	*Reference*
Yes	9.85 (8.58–11.30)	7.50 (6.87–9.20)	8.02 (5.21–12.35)	7.94 (7.22–8.72)	8.91 (8.06–9.82)
Age group					
≤ 39	NA	NA	NA	NA	*Reference*
40–44	NA	NA	NA	NA	3.40 (3.57–4.05)
≥ 45	NA	NA	NA	NA	4.72 (4.05–5.50)
Gestational age					
At term, 37–42 weeks	*Reference*	*Reference*	*Reference*	*Reference*	*Reference*
Post term, > 42 weeks	0.63 (0.23–1.72)	0.92 (0.49–1.75)	1.65 (0.19–14.31)	0.90 (0.54–1.50)	0.86 (0.51–1.44)
Preterm, 32–36 weeks	1.37 (1.14–1.65)	1.20 (1.00–1.43)	0.95 (0.55–1.66)	1.26 (1.12–1.43)	1.26 (1.12–1.43)
Very preterm, < 32 weeks	1.57 (0.93–2.64)	1.92 (1.15–3.19)	0.13 (0.02–0.93)	1.47 (1.03–2.10)	1.60 (1.11–2.29)
Birthweight					
Normal birthweight, ≥2500 g	*Reference*	*Reference*	*Reference*	*Reference*	*Reference*
Low birthweight, 1500 g - 2499 g	1.01 (0.81–1.25)	0.90 (0.72–1.11)	1.62 (0.88–2.99)	1.02 (0.88–1.17)	0.98 (0.85–1.14)
Very low birthweight, < 1500 g	0.81 (0.56–1.45)	0.59 (0.34–1.02)	4.82 (0.67–34.83)	0.82 (0.56–1.20)	0.70 (0.47–1.04)
Size for gestational age					
AGA^1^	*Reference*	*Reference*	*Reference*	*Reference*	*Reference*
SGA^2^	1.35 (0.97–1.88)	0.84 (0.64–1.10)	1.54 (0.69–3.42)	1.16 (0.96–1.42)	1.02 (0.83–1.24)
LGA^3^	1.27 (0.98–1.64)	1.08 (0.88–1.32)	1.70 (0.92–3.16)	1.22 (1.05–1.43)	1.17 (1.00–1.37)

^a^Adjusting for all variables presented in Table 4

^1^Appropriate for gestational age

^2^Small for gestational age

^3^Large for gestational age

The multivariate analyses (covariates included were civil status, method of conception, mother’s BMI in early pregnancy, and mother’s age when giving birth) revealed that being single or underweight, having an ART treatment, and being older were all factors negatively related to birthweight, child’s length at birth as well as gestational age (Table **7**). In the multiple logistic regression models of pregnancy and delivery complications women of a higher age and BMI were found to have an increased odds ratio for hypertensive disease during pregnancy, gestational diabetes, pre-eclampsia/eclampsia, and having a child with registered health problems during the neonatal period (Table **7**). Alternative multivariate models where twinning of the child was excluded were also considered. However, since twins are often born preterm and/or with a lower birth weight and that older women are more likely to have undergone an ART-treatment to achieve a pregnancy, which in itself is considered a risk factor for twinning, low birthweight, and prematurity, it was decided to keep the variable twinning in the final models. In a sub-group analysis of multiparous women, the findings of increased the risks for negative pregnancy and delivery outcomes among older women where verified (data not shown).

**Table 7:** Multivariate analysis on pregnancy complications and pregnancy outcomes

	Birthweight^a^	Gestational age^a^	Hypertension^b^	Diabetes^b^	(Pre)eclampsial^b^	Unhealty child^b^	Survival^b^
	B (95% CI)	B (95% CI)	OR (95% CI)	OR (95% CI)	OR (95% CI)	OR (95% CI)	OR (95% CI)
Age when giving birth							
≤ 39	*Reference*	*Reference*	*Reference*	*Reference*	*Reference*	*Reference*	*Reference*
40–44	−38.44 (−46.36 - -30.53)	−0.12 (− 0.15 - -0.10)	1.58 (1.39–1.79)	1.97 (1.79–2.18)	1.52 (1.40–1.64)	1.22 (1.18–1.26)	1.62 (1.20–2.19)
**≥** 45	−97.49 (−123.94 - -71.05)	−0.39 (− 0.47 - -0.30)	2.00 (1.43–2.80)	2.90 (2.27–3.70)	2.49 (2.05–3.02)	1.43 (1.29–1.59)	2.28 (1.05–4.93)
Civil status							
Single status at registration	−85.60 (−99.15 - -72.06)	−0.09 (− 0.14 - -0.05)	0.72 (0.57–0.92)	1.03 (0.87–1-22)	1.02 (0.89–1.16)	1.13 (1.07–1.20)	1.32 (0.81–2.12)
Married/cohabiting	*Reference*	*Reference*	*Reference*	*Reference*	*Reference*	*Reference*	*Reference*
ART							
Yes	−31.26 (−48.89 - -13.63)	−0.20 (−0.26 - -0.14)	1.12 (0.89–1.42)	1.11 (0.90–1.37)	1.53 (1.34–1.75)	1.46 (1.36–1.56)	1.63 (0.94–2.82)
No	*Reference*	*Reference*	*Reference*	*Reference*	*Reference*	*Reference*	*Reference*
Parity							
Primiparous	*Reference*	*Reference*	*Reference*	*Reference*	*Reference*	*Reference*	*Reference*
Multiparous	158.85 (149.87–167.84)	−0.06 (−0.10 - -0.04)	0.50 (0.44–0.57)	1.22 (1.07–1.40)	0.42 (0.38–0.45)	0.70 (0.67–0.72)	1.16 (0.80–1.70)
Twin							
No	*Reference*	*Reference*	*Reference*	*Reference*	*Reference*	*Reference*	*Reference*
Yes	−3.88 (−3.97- -3.80)	−3.09 (−3.15- -3.03)	1.48 (1.14–1.92)	1.042 (0.80–1.31)	4.93 (4.40–5.53)	4.89 (4.57–5.24)	8.67 (6.10–12.32)
Mother’s BMI (registration)							
Normal	*Reference*	*Reference*	*Reference*	*Reference*	*Reference*	*Reference*	*Reference*
Underweight	− 232.52 (− 262.07 - -202.97)	−0.34 (−0.43 - −0.24)	0.60 (0.28–1.26)	0.92 (0.49–1.74)	0.71 (0.47–1.08)	1.09 (0.96–1.24)	–
Overweight	81.97 (73.57–90.37)	0.03 (−0.02–0.03)	1.66 (1.44–1.91)	2.21 (1.94–2.52)	1.61 (1.48–1.76)	1.21 (1.17–1.26)	1.25 (0.90–1.74)
Obese	129.14 (118.47–139.80)	-0-11 (− 0.14 - -0.07)	2.89 (2.49–3.36)	7.18 (6.37–8.10)	2.88 (2.62–3.17)	1.66 (1.59–1.73)	1.50 (1.02–2.22)

^a^Linear regression, adjusted for all variables in Table 5

^b^Logistic regression, adjusted for all variables in Table 5

## Discussion

The results reveal that women of advanced age were more often single, had used ART, and had adverse obstetric outcomes more often than did younger women. The neonates born to women of advanced age were more likely to have health problems including being underweight or SGA, having lower a Apgar, and having other additional health problems during the first week.

The strength of the study is the availability of register data on all the women who gave birth in Sweden during the study period and medical data that were reported in a standardized form, thus minimizing recall bias. One limitation in this study is that we had no information about the ART method used. It might be that for single women of relatively advanced age the use of embryo donation is the method used, whereas for cohabiting women of advanced age it might be more likely that they had used their partner’s sperm and an oocyte donation. In the MBR register there are also limited data on the women’s health status. ART treatment may have been underreported in all study groups; we have not been able to control for this through examination of registers on IVF pregnancies done in Sweden. Also, among women who have received treatment abroad underreporting is likely since there was no obligation to report ART to the midwife and obstetricians in the antenatal setting.

Our results correspond with results from other studies on older women’s health status and obstetric outcomes [7, 10, 12]. The reason for these outcomes might be multifactorial. One important factor might be the use of ART and the other factor the woman’s biological age. The woman’s fecundity clearly decreases with increasing age, generally more noticeably a few years after age 30, to cease about 10 years before menopause [18].

There is only limited information on the use of ART for women in advanced age groups and the information is often available only through case reports in which there is no information on the specific technique used [19].

The risks for the mother and the neonate with gamete donation and embryo donation are not fully understood and have not yet been thoroughly investigated. There seems to be a consensus that the risk for women to have pregnancy complications such as preeclampsia and bleeding and the risk for the neonate to be born prematurely or with low birth weight is of considerable importance [20, 21]. Having premature and small for gestational children might also affect the mother and family mentally, as well as economically and socially, both in long-term as well as in short-term [22, 23].

For women who have given birth at a relatively advanced age there is still no consensus in the medical literature on the specific age or age range when the risk elevates significantly; some claim that only after 40 years of age there are risk of clinical significance but others report that the risk is already clinically significant at 35 years [24, 25]. In a Norwegian cohort of over 40,000 women who all answered a questionnaire at around gestational week 17, the authors found that women older than 38 years experienced problems related to physical aging including hypertension, back and shoulder problems, and diabetes. Furthermore, these women were more likely to have mental health problems than younger women [26].

Women in general might not be aware of obstetric and delivery risks for older women, and women who seek treatment abroad with ART and with donated gametes might be especially optimistic. It also seems possible that society at large and some medical practitioners tend to be overly affirmative in an effort not to discriminate against older women. This may even be true of personnel at clinics offering treatment. For women treated in other settings, for example abroad in private clinics, there may be age limits different from those in Sweden or even no age limits at all. There are other risks related to ART treatment in women of advanced age who have been treated abroad such as undetected illnesses both mental and physical, since not all clinics have access to medical records and thus have a limited medical history or have access to complete medical history and ongoing illnesses. Some women may themselves be unaware of the consequences or may want to have a child no matter what risks.

The women who were single when giving birth seem to represent a group of women that have more medical problems during pregnancy and delivery than women who are not single. The findings in this study indicate that women who are of very advanced age (above 45 years of age), single and had used ART are most likely to be women who have gone abroad to have their treatment since they cannot be treated in Sweden.

The increased risks for older women giving birth have important clinical implications and professionals need to be fully aware of the risks they expose the older women to by letting them go through ART. For children who were born preterm and SGA there may be future long-term health problems that will require medical attention and care. The development and needs of children born in families where the parents are of advanced age have not yet been well studied.

## Conclusions

In conclusion, the results from this study show that there is a substantial need to inform older, as well as younger, women about reproductive issues. This includes guidelines, both in general and in clinical practices, on becoming pregnant and whether to use or not to use ART. Women who are in the later stages of their reproductive life are at great risk for having children who are preterm and SGA. This may have long-term consequences not only for the children but also for the mothers. IVF clinics need to show a great medical awareness when offering older women treatment with their own gametes and with donated sperm and oocytes.

Society must also be more aware of the risks and to be more willing to discuss reproductive problems associated with advanced maternal age. For women and children, the need for future care is also a medical and societal matter that needs to be given more attention so that approaches are developed to serve these families both in the long as well as the short term.
